# Functional implications of Neandertal introgression in modern humans

**DOI:** 10.1186/s13059-017-1181-7

**Published:** 2017-04-03

**Authors:** Michael Dannemann, Kay Prüfer, Janet Kelso

**Affiliations:** Max Planck Institute for Evolutionary Anthropology, Deutscher Platz 6, Leipzig, 04103 Germany

**Keywords:** Neandertal introgression, Gene expression regulation, Protein sequence variation, Human evolution

## Abstract

**Background:**

Admixture between early modern humans and Neandertals approximately 50,000–60,000 years ago has resulted in 1.5–4% Neandertal ancestry in the genomes of present-day non-Africans. Evidence is accumulating that some of these archaic alleles are advantageous for modern humans, while others are deleterious; however, the major mechanism by which these archaic alleles act has not been fully explored.

**Results:**

Here we assess the contributions of introgressed non-synonymous and regulatory variants to modern human protein and gene expression variation. We show that gene expression changes are more often associated with Neandertal ancestry than expected, and that the introgressed non-synonymous variants tend to have less predicted functional effect on modern human proteins than mutations that arose on the human lineage. Conversely, introgressed alleles contribute proportionally more to expression variation than non-introgressed alleles.

**Conclusions:**

Our results suggest that the major influence of Neandertal introgressed alleles is through their effects on gene regulation.

**Electronic supplementary material:**

The online version of this article (doi:10.1186/s13059-017-1181-7) contains supplementary material, which is available to authorized users.

## Background

Some archaic alleles have been shown to confer an adaptive advantage for modern humans, and some of the most striking candidates for adaptive introgression from Neandertals are associated with traits related to environmental adaptation, including immunity and high altitude and skin and hair physiology in non-Africans [1–5]. However, recent studies have explored the effects of selection on archaic variants and have suggested that the depletion of archaic ancestry around functional elements in the genomes of present-day people reflects widespread purifying selection against archaic variants [5–7]. Selected variants can exert their effect by modifying gene expression or by changing the amino acid sequence [8–12]. Although both mechanisms have been described for introgressed alleles, and it has been suggested that regulatory changes are likely to have a larger impact [8], the relative contribution of each mechanism remains unknown.

Neandertal alleles that introgressed into modern humans are likely to be those that were at an appreciable frequency in the Neandertal population and are therefore likely to be older than their generally low frequency in modern humans suggests. To determine whether they have disproportional functional impact compared to non-archaic variants of matched frequency, we identify introgressed Neandertal alleles in present day people that affect either protein coding potential or gene regulation and compare their effects on molecular phenotypes to non-introgressed alleles of a similar frequency. We are able to show that some of the introgressed alleles that modify the molecular phenotype are responsible for phenotypic variation in modern humans. We also study changes in frequency of these alleles to understand the selective pressures under which they have evolved in recent modern human history.

## Results

We defined putatively introgressed alleles as those that differ between the Altai Neandertal and all Yoruba individuals in the 1000 Genomes [13] (“Methods”) and that overlap with the previously published Neandertal introgression map for modern humans [5] (“Methods”). Although it is possible that a subset of sites are mis-labeled due to error, incomplete lineage sorting, and the divergence between introgressing and sequenced Neandertal genomes [14], this approach enriches for alleles of Neandertal origin. We then annotated introgressed alleles that modify amino acid sequences [15] and tested alleles within 50 kb of genes for their association with gene expression in multiple human tissues [16].

### Impact of introgressed alleles on protein sequences

**Fig. 1:**
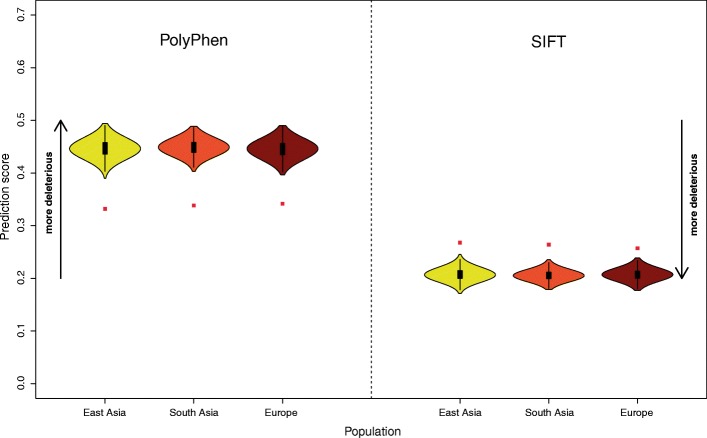
Functional impact of Neandertal non-synonymous alleles. *Left*: Average PolyPhen2 scores for archaic non-synonymous alleles in East Asians, South Asians, and Europeans (*red squares*). These averages are compared to averaged Polyphen2 scores for 1000 frequency matched sets of non-archaic non-synonymous alleles (*yellow*, *orange*, and *brown violin plots*). Polyphen2 scores range from 0–1 with higher scores associated with increased deleteriousness. *Right*: Average SIFT scores for archaic non-synonymous alleles in East Asians, South Asians, and Europeans (*red squares*). These averages are compared to averaged SIFT scores for 1000 frequency matched sets of non-archaic non-synonymous alleles (*yellow*, *orange*, and *brown violin plots*). SIFT scores range from 0–1 with lower scores associated with increased deleteriousness

We detected a total of 930 alleles that result in non-synonymous changes in present-day Eurasians (Europeans 701, East Asians 740, South Asians 841; “Methods”) and compared the predicted effect of these changes using SIFT and PolyPhen2 [17, 18] to the effects of a set of frequency-matched, non-synonymous non-archaic alleles. SIFT and PolyPhen2 provide two approaches to predict the functional impact of amino acid substitutions based on their proximity to functional domains, the physico-chemical properties of the substitution, and evolutionary or protein family conservation. We found that non-synonymous archaic alleles are predicted to have less effect (as measured by the deleteriousness scores) than non-synonymous non-archaic alleles (all *P* < 0.001; Fig. 1; “Methods”).

### Impact of introgressed alleles on gene expression

To identify changes in gene expression that are potentially mediated by introgressed alleles we used genotype and expression data for 48 tissues and 450 individuals (Additional file 1: Table S1) provided by the GTEx consortium [16].

To identify loci that are potentially of archaic origin we cluster alleles in high linkage disequilibrium (r^2^ > 0.8) and then select an archaic-like tag allele for each of the identified archaic loci (between 6118 and 9887 loci per tissue; “Methods”; Additional file 1: Table S1). Similarly, we identify non-archaic loci as those where none of the alleles in linkage disequilibrium (LD) are of archaic origin and select for each of these non-archaic loci a random tag allele (between 919,090 and 1,640,104 loci per tissue). We then identified between 4322 and 6008 expressed genes within 50 kb of archaic loci and between 16,857 and 17,044 genes within 50 kb of non-archaic loci that are potentially regulated by the archaic or non-archaic variants, respectively. For each tissue we computed genotype-expression association (GEA) by correlating the genotypes of the tag alleles with the expression of nearby gene/s (“Methods”). Neandertal introgression results in longer haplotypes—or at least haplotypes of a length consistent with the introgression time—that are therefore more likely to contain more alleles in LD (lengths of archaic and non-archaic loci shown in Additional file 2: Figure S1). Picking the best association may therefore be biased as there are more potentially associated alleles to choose from in archaic loci than in non-archaic loci. To avoid artificially inflating associations for archaic loci we pick a random allele to represent both archaic and non-archaic loci.

We identified loci in each tissue where an archaic allele was significantly associated with an expression change (false discovery rate (FDR) <0.05; “Methods”, Additional file 1: Table S1). The number of significant archaic loci (between 1 and 211) was highly correlated with the number of samples in the tissue (rho = 0.93, *P* = 8.9e-22), indicating that our power to detect significant GEAs is dependent on the number of individuals for which we have data in a given tissue. This difference in power and the variation of expression constraint between tissues [19, 20] made it difficult for us to directly compare results between tissues. However, we observed a significant excess of low *P* values among the top 5% of genes showing differential expression that is related to Neandertal ancestry, and therefore defined the top 5% of genes associated with archaic loci to be significant GEAs for each tissue (Additional file 2: Figure S2). We found that most GEAs are detected in only one tissue (27% of GEAs) or are shared between a small number of tissues (79% of significant GEAs are shared between four or fewer tissues; “Methods”). We caution that these results are sensitive to differences in expression variation between tissues.

**Fig. 2:**
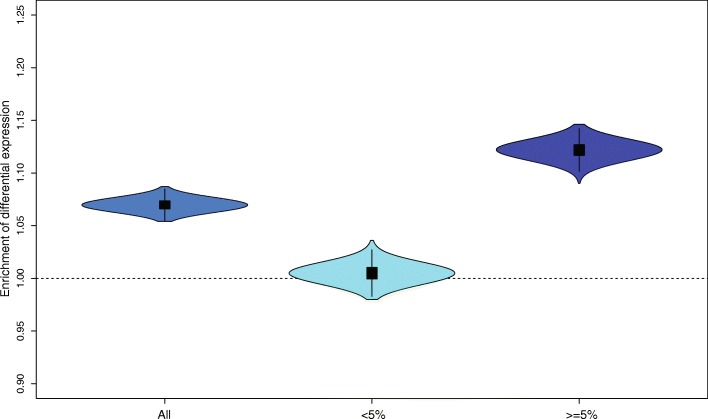
Enrichment of significant GEA archaic loci compared to non-archaic loci across all tissues. Each violin plot shows the distribution of the ratio between the number of significant archaic GEAs and 1000 samples of significant non-archaic GEAs for all archaic loci (*blue*), the subset of archaic loci with a Neandertal allele frequency <5% (*dark blue*), and the subset of archaic loci with a Neandertal allele frequency ≥5% (*light blue*)

To determine whether introgressed alleles contribute significantly to expression variation, we compared GEAs of our archaic tag alleles to GEAs of a set of frequency-matched non-introgressed tag alleles. Selecting frequency-matched archaic and non-archaic alleles ensures that we have similar power to detect expression differences. For each tissue we computed the number of archaic loci with a significant GEA (top 5% *P* values). When pooling all tissues, we found that a significantly higher number of archaic loci were associated with changed gene expression compared to non-archaic loci (*P* < 0.001; “Methods”; Fig. 2). When testing tissues individually, 23 of 48 tissues had significantly more archaic loci associated with expression changes than non-archaic loci (FDR < 0.05; “Methods”; Additional file 1: Table S2).

Interestingly, there was no enrichment for differential expression associated with archaic loci at lower frequencies (archaic allele frequency <5%) when all tissues were combined (*P* < 0.28; Fig. 2; “Methods”), and in only 10 of the 48 tissues individually (FDR <0.05; “Methods”; Additional file 1: Table S2), suggesting that the signal is mainly driven by higher-frequency introgressed alleles. Indeed, archaic alleles with a frequency ≥5% are enriched near genes that are differentially expressed in the pooled tissue set (*P* < 0.001), and also near genes that are differentially expressed in many individual tissues (26/48 tissues with FDR <0.05).

### Frequency changes in introgressed alleles

In order to study recent changes in the frequencies of introgressed alleles in modern humans, we used selection scores from a catalog of sites for which allele frequency data in a set of modern humans ranging from around 8000 years to the present day are available [21]. We extracted selection scores from this set for 80 archaic non-synonymous alleles in Europeans, 79 in South Asians, and 76 in East Asians. Three of these non-synonymous archaic alleles are shared between all populations and all three have significantly decreased in frequency since 8000 years ago, which is surprising given that there was an overall tendency for the remaining set of 77 non-synonymous archaic alleles to increase in frequency (51/77 show a non-significant increase, Fisher’s exact test *P* = 0.04). When comparing archaic non-synonymous alleles to frequency-matched, non-archaic non-synonymous alleles we found that a similar proportion of the non-archaic alleles show a significant frequency shift over time (Europe *P* = 0.16, East Asia *P* = 0.09, South Asia *P* = 0.15; FDR = 0.16 for all populations; “Methods”). However, a higher fraction of non-archaic alleles increased significantly in frequency in all populations than was the case for the archaic alleles (Europe *P* = 0.04, East Asia *P* = 0.03, South Asia *P* = 0.05; FDR = 0.05 for all populations; “Methods”). We also compared the frequency changes in non-synonymous archaic alleles to changes in synonymous archaic alleles. We identified 743 synonymous archaic alleles in East Asians, 886 in South Asians, and 766 in Europeans and extracted selection scores from Mathieson et al. [21] for 37, 44, and 44, respectively. Since there was no difference in the distributions of archaic allele frequencies for synonymous and non-synonymous sites (*P* = 0.63 Mann-Whitney-U test; “Methods”) we compared the proportion of significant selection scores for archaic synonymous and non-synonymous alleles directly. We found five synonymous archaic alleles in all three populations that showed significant frequency changes; two of them increased in frequency over time, while three decreased. The fraction of alleles that increased significantly is not statistically significantly higher than the fraction of archaic non-synonymous alleles (Europe and South Asia *P* = 0.13, East Asia *P* = 0.05, FDR = 0.13 for all three populations; “Methods”). Our results suggest that archaic non-synonymous variants decreased in frequency more often than expected compared to non-archaic non-synonymous variants and show similar frequency changes to those seen among archaic synonymous variants. This is consistent with similar or slightly more negative selection on archaic amino acid-changing variants compared to non-archaic amino acid-changing variants. However, we note that these results are based on very few alleles and that additional data would be useful to confirm these observations.

**Fig. 3:**
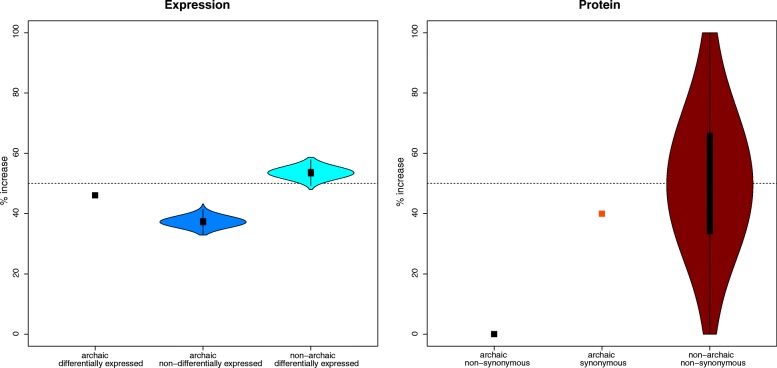
Direction of change for alleles showing significant frequency changes in recent modern human history. *Left*: The percentage of archaic alleles showing association with differential expression in at least one tissue that increase in frequency, and comparison to two sets of frequency matched alleles: (i) archaic alleles not associated with differential expression; and (ii) non-archaic alleles that are associated with differential expression. *Right*: The percentage of non-synonymous archaic alleles that increase in frequency compared to (i) synonymous archaic alleles and (ii) frequency-matched, non-synonymous non-archaic alleles

We show above that high frequency archaic alleles (≥5%; Additional file 1: Table S2) seem to contribute more to differences in gene expression, suggesting that introgressed variants in regulatory regions may have increased in frequency in non-Africans. To determine the extent to which archaic alleles that modify expression have changed in frequency in recent human history, we first assigned to each significantly associated archaic locus (GEA) the most significant selection score for an archaic allele (lowest *P* value) within the locus. For comparison, we selected as many frequency-matched non-archaic loci that are equally strongly associated with differential expression and assign to these the most significant selection score for the locus (“Methods”). We find that archaic loci associated with differential expression in a pooled set of all tissues show significant frequency changes more often than frequency-matched non-archaic loci (*P* < 0.001; “Methods”; Additional file 1: Table S3). Similarly, when we compare archaic loci associated with differential expression to frequency-matched archaic loci that are not associated with differential expression, we find that expression-changing archaic loci show more significant frequency changes than archaic loci that do not change expression (*P* < 0.001; Additional file 1: Table S3; “Methods”). We show that archaic alleles associated with differential expression significantly change their frequency more often than expected, and that 54% of archaic alleles associated with differential expression decreased significantly in frequency, which was less than observed in the matched sets of archaic alleles with no expression differences (57–67%, *P* < 0.001; “Methods”). In contrast, matched non-archaic alleles associated with expression changes showed an average 53% increase in allele frequencies (ranging between 48 and 59%, *P* < 0.001; Fig. 3; Additional file 2: Figure S3; “Methods”). We note that the archaic alleles associated with expression changes were ascertained in pre-dominantly Europeans from the GTEx panel and therefore they may not be representative of the regulatory effect in other non-African populations.

**Fig. 4:**
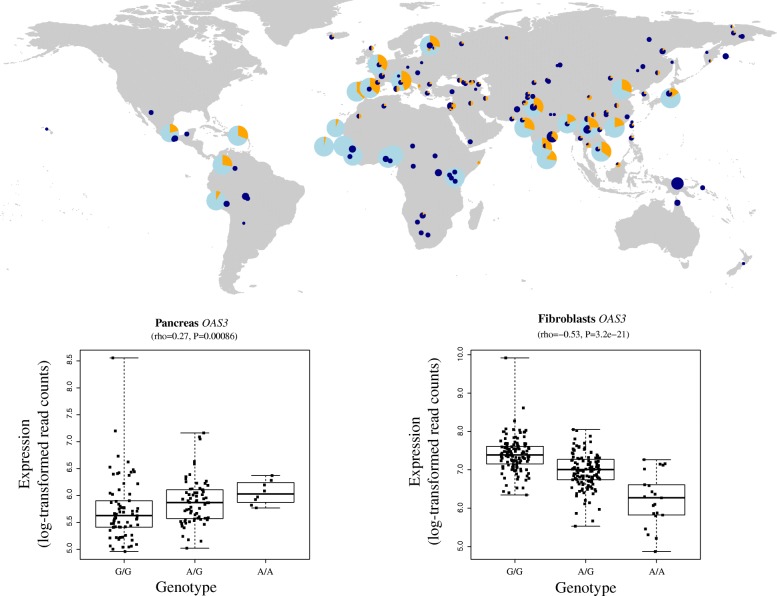
Global frequency distribution of archaic alleles at the OAS gene cluster and differential expression for *OAS3*. The frequency of the archaic locus (*orange*) spanning the OAS gene cluster in present-day human populations from the 1000 Genomes phase III (*light blue*) and Simons Genome Diversity Project (*dark blue*) datasets are shown in the *upper panel*. The sizes of the *pie charts* are proportional to the number of individuals in each population. The *lower panel* shows the genotype-dependence of expression (log-transformed read counts) of *OAS3* in pancreas (*left*) and fibroblasts (*right*). The expression distributions for both homozygote and the heterozygote states are shown as box plots and expression in each individual is plotted as a *black square*. The Spearman correlation coefficient and the corresponding *P* value for both tissues are shown above each graph. The introgressed Neandertal allele is the “*A*” (chr12:113366899)

Overall, non-synonymous archaic alleles and archaic alleles associated with expression changes have decreased in frequency over the past ~8500 years. However, we identified four loci where the archaic alleles associated with differential expression show large increases in frequency over time (Additional file 1: Table S4; “Methods”). Among these are introgressed alleles modifying expression of the *OAS1*/*OAS2*/*OAS3* genes, which are involved in innate immunity. Elevated introgression has already been reported [22] for these genes and we find that the expression-changing alleles exhibit the most extreme change in frequency (corrected *P* value = 1.14 × 10^−8^, genome-wide rank 314 across all SNPs) with archaic alleles reaching frequencies of 28–44% in present-day Europeans and 16–35% in present-day Asians (Additional file 1: Table S4; Additional file 2: Figure S4). Interestingly, we observe tissue-specific differences in the effects of the archaic alleles on gene expression (Fig. 4). For example, the *OAS1*, *OAS2*, and *OAS3* genes are in the top 5% GEA loci in four, two, and eight tissues, respectively (Additional file 1: Table S4). Archaic alleles in *OAS1* are associated with higher expression in subcutaneous adipose tissue and sun-exposed skin, while higher expression in thyroid and pancreas and vagina is associated with archaic alleles in *OAS2* and *OAS3*, respectively. In contrast, individuals carrying archaic alleles show down-regulation of *OAS1* and *OAS3* in esophagus mucosa and spleen, and individuals carrying archaic alleles show down-regulation of *OAS2* in fibroblasts and *OAS3* in fibroblasts as well as three brain regions (hippocampus, putamen, and caudate nucleus; Fig. 4). The tissue-specific effects of these archaic alleles suggest that they may be functionally relevant.

### Phenotype associations

To determine whether archaic alleles influence particular phenotypes in present-day humans, we identified from among the top 1% of archaic GEAs 14 loci (*P* value <10^-8^) where an archaic allele in the locus matches an allele previously reported to be significantly associated in one or more genome-wide association studies, and a further six archaic alleles that match the most significant GWAS association in one or more studies (1 × 10^−8^ < *P* value < 1 × 10^−5^; Additional file 1: Table S5). Similarly, we identify six non-synonymous archaic alleles with significant GWAS associations (*P* value <10^−8^) and three that match the most significant GWAS association in one or more studies (1 × 10^−8^ < *P* value < 1 × 10^−5^). It is difficult to construct a meaningful enrichment test that accounts for confounding factors present in this collection of GWAS studies, but we note that for both non-synonymous and regulatory changes there are a number of categories associated with metabolic pathways and with immunity, as well as a number of neurological associations. Interestingly, we are now able to elucidate the molecular impact of three of the variants reported in Sankararaman et al. [5]. The introgressed haplotype at *ZNF365* (*rs7076156*, hg19, chr10:64415184) carries a non-synonymous allele associated with risk of Crohn’s disease with frequencies up to 32% in Europeans [23]; Additional file 1: Table S6). In addition, an archaic allele on chromosome 11 (*rs1834481*, hg19, chr11:112023827) is associated with reduced expression of *IL18* in multiple tissues, including the pancreas, and *IL18* levels/markers of inflammatory response in two GWAS studies [24, 25] and present in Europeans at frequencies up to 24%. Another regulatory archaic variant, *rs12531711* (hg19, chr7:128617466), modifies the expression of *TNPO3* in brain and is associated with multiple auto-immune phenotypes [26–35] (Additional file 1: Table S6).

Further, we find a GEA locus which includes *rs17612333* (hg19, chr4:169330384) for which the archaic allele is associated with reduced expression of *DDX60L* in subcutaneous adipose tissue and an increased body mass index (BMI) in Native Americans [36]. The archaic allele is present at frequencies between 13 and 21% in European populations and shows similar frequencies in Asians (8–15%; Additional file 1: Table S6; Additional file 2: Figure S4). A second archaic GEA locus that is associated with changed expression of *COL13A1* includes *rs17497526* (hg19, chr10:71580120), an allele associated with Parkinson’s disease risk in North Americans [37]. The risk allele is likely of archaic origin, and individuals carrying the archaic allele in the GTEx dataset show significantly lower expression of *COL13A1* in the cerebellum compared to individuals without the introgressed archaic allele. The archaic allele is more prevalent in European populations (7–15%) than in Asians (2–11%), with the frequency in East Asian populations substantially lower (2–5%; Additional file 1: Table S6; Additional file 2: Figure S4). It is intriguing that some studies that have suggested a lower prevalence of Parkinson’s disease in Asia [38], opening the question of whether the archaic introgression may contribute to Parkinson’s risk in Europeans.

## Discussion

Recent studies have shown that Neandertal alleles are depleted in more constrained, functional regions of the genome and that, on average, selection has acted to remove introgressed Neandertal alleles from the modern human population [5, 7, 39]. Despite these general patterns, a number of instances of adaptive introgression have been described, generally affecting systems that influence immune and metabolic phenotypes [1, 5, 10, 22, 40, 41]. The mechanisms by which these adaptive alleles act have not been widely explored. We show here that Neandertal alleles contributing to variation in protein sequences and expression have, in general, decreased in frequency during recent modern human history. This is particularly the case near protein coding genes, and is consistent with negative selection on Neandertal DNA in modern humans [5, 6, 7, 39]. However, we cannot exclude the possibility that some of the introgressed variants also experienced negative selection in Neandertals prior to admixture. The difference in deleteriousness between frequency matched archaic and non-archaic alleles in modern humans may therefore reflect a mixture of these two effects.

The surviving Neandertal DNA seems to have contributed significantly to variation in gene expression in modern humans compared to other non-introgressed variants. Although the enrichment for differential expression linked to archaic ancestry is only between ~5 and 10% for all tissues, the fact that there are thousands of archaic alleles across the genome means that the expression of several hundred genes is potentially affected. We also see that higher frequency archaic variants contribute significantly more to gene expression changes than lower frequency archaic variants, suggesting that at least some of the archaic alleles that modify gene expression may have been driven to higher frequencies by positive selection, and supporting the idea that changes in gene expression are likely to have important adaptive effects in humans [42].

## Conclusions

We provide evidence that changes in both protein sequence and in expression introduced by Neandertal DNA have phenotypic consequences for present-day people. However, our results indicate that introgressed archaic DNA is likely to exert a larger effect through changes in gene regulation than through modifications to protein sequences.

## Methods

### Genotype data and assignment of putative introgressed variants

We used genotype data for 450 individuals for whom expression data are also available (GTEx [43] version 6; Additional file 1: Table S1). Of a total of 10,531,619 SNPs in GTEx, we used 7,400,760 that are located 50 kb up- and downstream of protein-coding genes (ENSEMBL: GRCh37) and showed variation between individuals. These SNPs were then assigned to each of the protein-coding genes that were located within 50 kb of the SNP.

Next, we clustered SNPs into two sets. The first set consisted of 105,046 putative introgressed Neandertal-like SNPs (aSNPs), which we defined as having (i) one fixed allele in Yoruba individuals of the 1000 Genomes project (phase III) [13], (ii) a different allele in a heterozygous or homozygous state in the genome of the Altai Neandertal [14] which segregates in out-of-African GTEx individuals, and (iii) overlap with confidently inferred regions of Neandertal-introgression in modern humans. These introgressed regions are required to have a Neandertal posterior probability greater than 0.9 and a length of at least 0.02 cM [5]. The second set contained 7,282,603 SNPs that are not likely to be of archaic origin, i.e., SNPs where the Neandertal-shared allele is also present in Yoruba individuals. We call these “non-introgressed alleles”. The remaining 13,111 SNPs that do not fall in either set were excluded from any further analyses (Additional file 1: Table S1).

### Expression data

We used expression data for multiple individuals from 48 tissues for which at least 50 individuals with available genotype data were provided by GTEx (Additional file 1: Table S1). Five tissues with 5–26 individuals were excluded (Additional file 1: Table S1). All protein-coding genes for which at least two of the individuals for the given tissue had a read count greater than zero were defined as expressed in this tissue and were used in the following analyses (Additional file 1: Table S1). We use this low cut-off to accommodate the low frequency (~2%) of typical Neandertal alleles. Read counts for all expressed genes in a tissue were then normalized between individuals using the R package DESeq2 [44].

### Computing genotype-dependent expression (GEA)

For all SNPs for which we had at least two genotypes with a minimum of two individuals each, we computed Spearman’s correlation between the genotype, encoded as 1 (homozygous reference allele), 2 (heterozygous), and 3 (homozygous alternative allele), and the normalized expression of the nearby gene(s).

### Clustering of alleles in high LD

We clustered sets of alleles in high LD. We used PLINK [45] and combined sets of alleles with an *r* ^2^ ≥ 0.8 into one locus (PLINK parameters --ld-window-r2 0.8 --ld-window 99999). For each set of linked alleles we assign one of two possible classes: Neandertal-like or non-archaic. A set of linked alleles was defined to be Neandertal-like if at least one allele is Neandertal-like (“Neandertal-like locus”). Sets of linked alleles without Neandertal-like alleles were defined to be non-archaic (“non-archaic locus”). It is possible for both sets that loci contain a single SNP if no other variant in high LD could be identified. For each set of linked alleles we chose a representative allele using two algorithms to select this allele: in Neandertal-like sets the representative allele was either (i) the Neandertal-like allele within the set with the most significant genotype-expression correlation or (ii) a random Neandertal-like allele. In the non-archaic the representative allele was either (i) the allele within the cluster with the most significant genotype-expression correlation or (ii) a random allele. Representative alleles defined according to the second criterion were used for statistical analysis comparing archaic and non-archaic loci in order to avoid differences in power. The sets defined by the first criterion were used for the GWAS comparison and for the comparison of top GEA loci between tissues (see following section "Contribution of archaic loci to differential expression" for details).

In total we obtained data for 1,652,478 to 3,002,785 gene loci per tissue (Additional file 1: Table S1).

### Contribution of archaic loci to differential expression

To quantify the extent of differential expression associated with archaic loci compared to non-archaic loci we selected in each tissue the GEAs for all archaic loci. We computed the empirical tissue-specific 5% quantiles on the corresponding tissue’s *P* value distributions. For each tissue we randomly selected the identical number of frequency-matched non-archaic loci, i.e., non-archaic loci with the same frequency distribution of their tag-alleles as the frequency of the Neandertal-like tag-alleles selected for the archaic loci. For each tissue we computed the number of random non-archaic loci with a smaller GEA *P* value than the empirical 5% quantile defined based on the archaic loci *P* value distribution. At random, we would expect that non-archaic alleles reach the 5% quantile cutoff as often as the archaic alleles. In order to compute statistical significance, we repeated the re-sampling of non-archaic alleles 1000 times. The proportion of samples with at least as many significant GEAs gives us an empirical *P* value for each tissue. To compute an empirical *P* value over all tissues, we sum over all tissues for archaic loci and 1000 random samples of non-archaic loci. We repeated these analyses for archaic loci with Neandertal allele frequencies greater than and equal to 5%, and archaic loci with a Neandertal allele frequency lower than 5%. We corrected the obtained tissue-wise *P* values for multiple testing using the Benjamini-Hochberg procedure [46]; the reported expression FDR values therefore account for all tissue-specific tests performed.

### Detecting non-synonymous Neandertal alleles and computing deleteriousness

To identify synonymous and non-synonymous variants in present-day non-African human populations (Europeans, East Asians, and South Asians) [13] we used the variant effect predictor software (vep [15]). We selected non-synonymous variants (vep ID missense_variant) and synonymous Neandertal-like alleles (vep ID synonymous_variant) at a frequency greater than zero and used our defined archaic allele set (“Methods”, paragraph 2) to define these as introgressed alleles or non-introgressed alleles.

### Comparing deleteriousness scores between Neandertal-like alleles and non-archaic alleles

To classify non-synonymous changes according to their potential impact on the protein we used two scores, PolyPhen2 and SIFT [17, 18]. For each of the three meta-populations, Europeans, East Asians, and South Asians, we computed the average SIFT and PolyPhen2 deleteriousness scores for all non-synonymous variants in each population. To compare deleteriousness scores for the Neandertal non-synonymous variants to the scores for non-archaic non-synonymous allele, we sampled 1000 sets of non-archaic, non-synonymous alleles that were frequency matched to the Neandertal non-synonymous variants and computed their average SIFT and PolyPhen2 deleteriousness scores. The distribution of average deleteriousness scores for matched non-synonymous variants is shown in Fig. 1.

### Assigning selection scores for expression-associated loci

To link selection scores to GEA loci, we computed for each tissue the archaic loci with the lowest 5% GEA *P* values. We then intersected SNPs in each locus with SNPs reported by Mathieson et al. [21] and assigned the lowest selection score *P* value to each locus. Loci with no overlapping SNP in the selection set were excluded. For the remaining archaic loci we generated 1000 frequency- and size-matched sets each of (i) archaic loci with a selection score and a GEA *P* value outside the lowest 5% of the GEA *P* value distribution and (ii) non-archaic loci with a selection score and with a GEA *P* value smaller than the 5% quantile GEA *P* value of the archaic loci GEA *P* value distribution in the corresponding tissue. Empirical enrichment *P* values were calculated as the proportion of random sets with a number of loci with a selection score *P* value <0.05 equal to or larger than the number of such loci with significant archaic GEAs with significant selection associations. We summed the number of loci with selection score *P* values <0.05 across tissues to assess significance over all tissues and corrected the resulting tissue-wise *P* values for multiple testing by the Benjamini-Hochberg procedure [46].

### Allele frequency changes for loci with significant selection scores

We compared the number of significant selection scores for Neandertal non-synonymous variants to two background sets: (i) frequency-matched, non-synonymous non-archaic alleles; and (ii) Neandertal synonymous variants.

For the first comparison we sampled 1000 sets of non-archaic non-synonymous alleles that were frequency matched to the Neandertal non-synonymous variants and computed the number of significant selection scores and the direction of the allele frequency change in each set. Due to the lower number of synonymous variants with selection scores, compared to non-synonymous Neandertal variants with selection scores, we were not able to implement a re-sampling strategy. However, since the distributions on synonymous and non-synonymous archaic variants do not differ (Mann-Whitney U test, *P* = 0.63), we compared the number of significant selection scores and the direction of their frequency changes directly in each population using Fisher’s exact test.

We compared the number of significant selection scores for Neandertal GEAs to two background sets: (i) frequency-matched non-archaic loci with differential expression; and (ii) frequency-matched archaic loci with no differential expression.

For both comparisons we sampled 1000 sets of (i) frequency-matched non-archaic loci with differential expression and (ii) frequency-matched archaic loci with no differential expression and computed the number of significant selection scores and the direction of the allele frequency change in each set.

### Overlap of archaic alleles associated with differential expression with modern human phenotype data

We queried GWASdb [47, 49–72] to identify Neandertal-like alleles present in the most significant GEA loci (top 1% in a tissue; Additional file 1: Table S4) or the set of non-synonymous Neandertal alleles (Additional file 1: Table S5). We required that the association be either significant genome-wide (GWAS *P* value <1 × 10^−8^) or the top candidate in the corresponding publication with a *P* value of 1 × 10^−5^ or lower.

### Confirming that the identified loci are of archaic origin

For each candidate non-synonymous archaic SNP we extracted the Neandertal-like locus on which this SNP occurs. For the selection candidates and the GEA candidates we used the associated locus. We then defined the length of the putative archaic haplotype for each locus as the length of the segment between the two most distant archaic SNPs. To determine whether these putative archaic haplotypes are longer than expected due to incomplete lineage sorting, we used the approach by Huerta-Sánchez et al. [3], applying the age of the Altai Neandertal based on two commonly used mutation rates (μ = 1 × 10^−8^ and 0.5 × 10^−8^) [1] and the average recombination rates at each locus [48] (Additional file 1: Tables S4 and S5).

## Additional files

Additional file 1: Supplementary tables. (PDF 401 kb)

Additional file 2: Supplementary figures. (PDF 719 kb)
