# Supplementary material for: Functional implications of Neandertal introgression in modern humans
Source: Genome Biol. 2017 Apr 3;18:61. doi: 10.1186/s13059-017-1181-7 (PMC5376702; doi:10.1186/s13059-017-1181-7)
Supplement: Supplementary file 1 — Supplementary tables. (PDF 401 kb) [file 13059_2017_1181_MOESM1_ESM.pdf]

Table S1.

Basic information for differential expression analysis.

The table summarizes for each tissue (column 1) the number of individuals with expression and genotype data used in this study (column 2), the number of expressed protein coding genes (column 3), the total number of GEAs (column 4), split into those GEAs of putative archaic origin (column 5) and non archaic origin (column 6).
The summary values for five tissues, which were not included in the analyses due to the low number of individuals with genotype and expression data, are shown in gray.

Tissue	#individuals with genotype and expression data	#expressed genes	#GEAs	#archaic GEAs	#non-archaic GEAs	#GEAs with FDR<0.05
Adipose - Subcutaneous	298	17861	2941843	17966	2916563	145
Adipose - Visceral (Omentum)	185	17750	2657262	17201	2633271	61
Adrenal Gland	126	17793	2416744	16011	2394611	60
Artery - Aorta	197	17864	2664761	17465	2640405	102
Artery - Coronary	118	17746	2347748	15867	2325985	31
Artery - Tibial	285	17826	2923186	17933	2897962	141
Bladder	11	17242	647976	2813	644181	0
Brain - Amygdala	62	17516	1652478	12827	1635084	1
Brain - Anterior cingulate cortex (BA24)	72	17571	1767299	13494	1748999	3
Brain - Caudate (basal ganglia)	100	17689	2149394	15140	2128759	3
Brain - Cerebellar Hemisphere	89	17510	1980670	14612	1960805	25
Brain - Cerebellum	103	17578	2126304	15225	2105493	59
Brain - Cortex	96	17751	2157798	14910	2137522	12
Brain - Frontal Cortex (BA9)	92	17631	2050757	14660	2030731	14
Brain - Hippocampus	81	17564	1936159	14068	1917038	8
Brain - Hypothalamus	81	17669	1881547	14301	1862158	6
Brain - Nucleus accumbens (basal ganglia)	93	17598	2058368	14850	2038185	17
Brain - Putamen (basal ganglia)	82	17553	1957252	14026	1938207	9
Brain - Spinal cord (cervical c-1)	59	17505	1679378	12343	1662756	2
Brain - Substantia nigra	56	17496	1671128	12070	1654879	3
Breast - Mammary Tissue	183	17885	2684410	17245	2660449	32
Cells - EBV-transformed lymphocytes	114	17834	2400234	15393	2379037	52
Cells - Transformed fibroblasts	272	17871	2912050	17887	2886945	138
Cervix - Ectocervix	6	16997	122202	414	121565	0
Cervix - Endocervix	5	16817	0	0	0	0
Colon - Sigmoid	124	17717	2254808	15955	2232770	24
Colon - Transverse	169	17858	2621241	16978	2597646	26
Esophagus - Gastroesophageal Junction	127	17693	2331961	16054	2309757	27
Esophagus - Mucosa	241	17879	2799387	17733	2774538	139
Esophagus - Muscularis	218	17892	2753676	17610	2729055	104
Fallopian Tube	6	16870	130360	306	129858	0
Heart - Atrial Appendage	159	17720	2564619	16859	2541277	63
Heart - Left Ventricle	190	17707	2631690	17259	2607609	90

Kidney - Cortex	26	17362	1200830	6988	1191532	0
Liver	97	17706	2121865	14947	2101569	13
Lung	278	17867	2898226	17870	2873083	88
Minor Salivary Gland	51	17541	1662903	11122	1647923	1
Muscle - Skeletal	361	17902	3002785	18088	2977243	172
Nerve - Tibial	256	17883	2888421	17832	2863402	162
Ovary	85	17737	2087757	14109	2068553	22
Pancreas	149	17817	2535747	16531	2512794	63
Pituitary	87	17665	1982091	14353	1962578	29
Prostate	87	17792	2090320	14434	2070737	13
Skin - Not Sun Exposed (Suprapubic)	196	17787	2731676	17306	2707490	67
Skin - Sun Exposed (Lower leg)	302	17870	2937061	17947	2911812	200
Small Intestine - Terminal Ileum	77	17673	1967634	13590	1949110	9
Spleen	89	17805	2168824	14218	2149336	20
Stomach	170	17867	2629336	17059	2605653	32
Testis	157	17996	2546546	16798	2523269	110
Thyroid	278	17853	2908123	17914	2882929	215
Uterus	70	17754	1916768	13138	1899000	3
Vagina	79	17782	2009721	13727	1990992	8
Whole Blood	338	17883	2984698	18045	2959259	97

Table S2.

Differential expression enrichment of archaic loci compared to non-archaic loci

Significant comparisons (FDR<0.05) are highlighted in yellow.

	all	<=5%	>5%
Adipose - Subcutaneous	P=0.028 (FDR=0.054)	P=0.652 (FDR=1)	P=<0.001 (FDR=0.003)
Adipose - Visceral (Omentum)	P=<0.001 (FDR=0.003)	P=0.021 (FDR=0.084)	P=<0.001 (FDR=0.003)
Adrenal Gland	P=<0.001 (FDR=0.003)	P=0.42 (FDR=0.712)	P=<0.001 (FDR=0.003)
Artery - Aorta	P=0.001 (FDR=0.003)	P=0.395 (FDR=0.712)	P=<0.001 (FDR=0.003)
Artery - Coronary	P=0.941 (FDR=0.982)	P=1 (FDR=1)	P=0.11 (FDR=0.155)
Artery - Tibial	P=0.689 (FDR=0.778)	P=0.999 (FDR=1)	P=0.002 (FDR=0.006)
Brain - Amygdala	P=<0.001 (FDR=0.003)	P=<0.001 (FDR=0.01)	P=0.247 (FDR=0.304)
Brain - Anterior cingulate cortex (BA24)	P=0.034 (FDR=0.063)	P=0.086 (FDR=0.275)	P=0.102 (FDR=0.153)
Brain - Caudate (basal ganglia)	P=0.608 (FDR=0.712)	P=0.953 (FDR=1)	P=0.073 (FDR=0.113)
Brain - Cerebellar Hemisphere	P=<0.001 (FDR=0.003)	P=0.182 (FDR=0.416)	P=<0.001 (FDR=0.003)
Brain - Cerebellum	P=<0.001 (FDR=0.003)	P=0.001 (FDR=0.01)	P=<0.001 (FDR=0.003)
Brain - Cortex	P=0.258 (FDR=0.375)	P=0.92 (FDR=1)	P=0.008 (FDR=0.018)
Brain - Frontal Cortex (BA9)	P=0.013 (FDR=0.03)	P=0.032 (FDR=0.118)	P=0.132 (FDR=0.176)
Brain - Hippocampus	P=0.553 (FDR=0.682)	P=0.888 (FDR=1)	P=0.16 (FDR=0.208)
Brain - Hypothalamus	P=0.345 (FDR=0.485)	P=0.284 (FDR=0.551)	P=0.478 (FDR=0.499)
Brain - Nucleus accumbens (basal ganglia)	P=0.001 (FDR=0.003)	P=0.017 (FDR=0.074)	P=0.007 (FDR=0.018)
Brain - Putamen (basal ganglia)	P=0.007 (FDR=0.02)	P=<0.001 (FDR=0.01)	P=0.493 (FDR=0.503)
Brain - Spinal cord (cervical c-1)	P=0.554 (FDR=0.682)	P=0.699 (FDR=1)	P=0.383 (FDR=0.433)
Brain - Substantia nigra	P=0.009 (FDR=0.024)	P=0.003 (FDR=0.024)	P=0.293 (FDR=0.343)
Breast - Mammary Tissue	P=<0.001 (FDR=0.003)	P=0.007 (FDR=0.038)	P=<0.001 (FDR=0.003)
Cells - EBV-transformed lymphocytes	P=<0.001 (FDR=0.003)	P=0.113 (FDR=0.339)	P=<0.001 (FDR=0.003)
Cells - Transformed fibroblasts	P=<0.001 (FDR=0.003)	P=<0.001 (FDR=0.01)	P=<0.001 (FDR=0.003)
Colon - Sigmoid	P=0.139 (FDR=0.222)	P=0.778 (FDR=1)	P=0.009 (FDR=0.019)
Colon - Transverse	P=<0.001 (FDR=0.003)	P=<0.001 (FDR=0.01)	P=0.013 (FDR=0.025)
Esophagus - Gastroesophageal Junction	P=<0.001 (FDR=0.003)	P=0.122 (FDR=0.344)	P=<0.001 (FDR=0.003)
Esophagus - Mucosa	P=0.012 (FDR=0.029)	P=0.268 (FDR=0.551)	P=0.008 (FDR=0.018)
Esophagus - Muscularis	P=<0.001 (FDR=0.003)	P=0.445 (FDR=0.712)	P=<0.001 (FDR=0.003)
Heart - Atrial Appendage	P=0.024 (FDR=0.048)	P=0.14 (FDR=0.367)	P=0.04 (FDR=0.066)
Heart - Left Ventricle	P=0.354 (FDR=0.485)	P=0.999 (FDR=1)	P=<0.001 (FDR=0.003)
Liver	P=0.039 (FDR=0.067)	P=0.153 (FDR=0.367)	P=0.04 (FDR=0.066)
Lung	P=0.401 (FDR=0.535)	P=1 (FDR=1)	P=<0.001 (FDR=0.003)
Minor Salivary Gland	P=0.876 (FDR=0.934)	P=0.995 (FDR=1)	P=0.193 (FDR=0.244)
Muscle - Skeletal	P=0.088 (FDR=0.146)	P=0.993 (FDR=1)	P=<0.001 (FDR=0.003)
Nerve - Tibial	P=0.038 (FDR=0.067)	P=0.431 (FDR=0.712)	P=0.007 (FDR=0.018)
Ovary	P=0.535 (FDR=0.682)	P=0.681 (FDR=1)	P=0.394 (FDR=0.433)

Pancreas	P=0.15 (FDR=0.232)	P=0.403 (FDR=0.712)	P=0.109 (FDR=0.155)
Pituitary	P=0.014 (FDR=0.031)	P=0.287 (FDR=0.551)	P=0.009 (FDR=0.019)
Prostate	P=0.697 (FDR=0.778)	P=0.993 (FDR=1)	P=0.031 (FDR=0.055)
Skin - Not Sun Exposed (Suprapubic)	P=1 (FDR=1)	P=1 (FDR=1)	P=0.637 (FDR=0.637)
Skin - Sun Exposed (Lower leg)	P=0.863 (FDR=0.934)	P=1 (FDR=1)	P=0.014 (FDR=0.026)
Small Intestine - Terminal Ileum	P=0.021 (FDR=0.044)	P=0.008 (FDR=0.038)	P=0.431 (FDR=0.46)
Spleen	P=0.173 (FDR=0.259)	P=0.217 (FDR=0.473)	P=0.262 (FDR=0.314)
Stomach	P=0.01 (FDR=0.025)	P=0.15 (FDR=0.367)	P=0.01 (FDR=0.02)
Testis	P=<0.001 (FDR=0.003)	P=0.008 (FDR=0.038)	P=<0.001 (FDR=0.003)
Thyroid	P=<0.001 (FDR=0.003)	P=0.085 (FDR=0.275)	P=<0.001 (FDR=0.003)
Uterus	P=0.002 (FDR=0.006)	P=0.004 (FDR=0.027)	P=0.065 (FDR=0.104)
Vagina	P=0.579 (FDR=0.695)	P=0.713 (FDR=1)	P=0.397 (FDR=0.433)
Whole Blood	P=0.994 (FDR=1)	P=1 (FDR=1)	P=0.125 (FDR=0.171)

Table S3.

Enrichment of archaic alleles with significant frequency changes associated with differentially expressed archaic loci

Significant comparisons (FDR<0.05) are highlighted in yellow.

	vs. non-DE archaic	vs. DE non-archaic
Adipose - Subcutaneous	P=<0.001 (FDR=0.004)	P=0.001 (FDR=0.01)
Adipose - Visceral (Omentum)	P=0.031 (FDR=0.06)	P=0.057 (FDR=0.124)
Adrenal Gland	P=<0.001 (FDR=0.004)	P=<0.001 (FDR=0.01)
Artery - Aorta	P=0.068 (FDR=0.117)	P=0.06 (FDR=0.125)
Artery - Coronary	P=<0.001 (FDR=0.004)	P=0.001 (FDR=0.01)
Artery - Tibial	P=0.001 (FDR=0.004)	P=0.003 (FDR=0.018)
Brain - Amygdala	P=0.203 (FDR=0.287)	P=0.205 (FDR=0.289)
Brain - Anterior cingulate cortex (BA24)	P=0.227 (FDR=0.294)	P=0.222 (FDR=0.296)
Brain - Caudate (basal ganglia)	P=0.297 (FDR=0.356)	P=0.412 (FDR=0.471)
Brain - Cerebellar Hemisphere	P=0.205 (FDR=0.287)	P=0.462 (FDR=0.516)
Brain - Cerebellum	P=0.403 (FDR=0.45)	P=0.606 (FDR=0.619)
Brain - Cortex	P=0.215 (FDR=0.287)	P=0.173 (FDR=0.259)
Brain - Frontal Cortex (BA9)	P=0.024 (FDR=0.058)	P=0.076 (FDR=0.14)
Brain - Hippocampus	P=0.96 (FDR=0.96)	P=0.878 (FDR=0.878)
Brain - Hypothalamus	P=0.021 (FDR=0.053)	P=0.091 (FDR=0.147)
Brain - Nucleus accumbens (basal ganglia)	P=0.364 (FDR=0.416)	P=0.328 (FDR=0.394)
Brain - Putamen (basal ganglia)	P=0.291 (FDR=0.356)	P=0.181 (FDR=0.263)
Brain - Spinal cord (cervical c-1)	P=<0.001 (FDR=0.004)	P=0.046 (FDR=0.105)
Brain - Substantia nigra	P=0.511 (FDR=0.557)	P=0.485 (FDR=0.529)
Breast - Mammary Tissue	P=0.027 (FDR=0.059)	P=0.064 (FDR=0.128)
Cells - EBV-transformed lymphocytes	P=0.125 (FDR=0.2)	P=0.086 (FDR=0.147)
Cells - Transformed fibroblasts	P=0.006 (FDR=0.019)	P=0.005 (FDR=0.018)
Colon - Sigmoid	P=0.004 (FDR=0.014)	P=0.017 (FDR=0.058)
Colon - Transverse	P=0.278 (FDR=0.351)	P=0.285 (FDR=0.351)
Esophagus - Gastroesophageal Junction	P=0.001 (FDR=0.004)	P=<0.001 (FDR=0.01)
Esophagus - Mucosa	P=<0.001 (FDR=0.004)	P=0.002 (FDR=0.014)
Esophagus - Muscularis	P=0.003 (FDR=0.011)	P=0.018 (FDR=0.058)
Heart - Atrial Appendage	P=0.634 (FDR=0.662)	P=0.588 (FDR=0.614)
Heart - Left Ventricle	P=0.135 (FDR=0.209)	P=0.263 (FDR=0.332)
Liver	P=0.03 (FDR=0.06)	P=0.035 (FDR=0.088)
Lung	P=0.015 (FDR=0.04)	P=0.032 (FDR=0.085)
Minor Salivary Gland	P=0.15 (FDR=0.225)	P=0.214 (FDR=0.293)
Muscle - Skeletal	P=<0.001 (FDR=0.004)	P=<0.001 (FDR=0.01)
Nerve - Tibial	P=0.009 (FDR=0.025)	P=0.004 (FDR=0.018)
Ovary	P=0.055 (FDR=0.098)	P=0.04 (FDR=0.096)
Pancreas	P=0.048 (FDR=0.089)	P=0.027 (FDR=0.076)
Pituitary	P=0.031 (FDR=0.06)	P=0.092 (FDR=0.147)
Prostate	P=0.523 (FDR=0.558)	P=0.398 (FDR=0.466)
Skin - Not Sun Exposed (Suprapubic)	P=0.001 (FDR=0.004)	P=0.005 (FDR=0.018)

Skin - Sun Exposed (Lower leg)	P=<0.001 (FDR=0.004)	P=0.005 (FDR=0.018)
Small Intestine - Terminal Ileum	P=0.74 (FDR=0.756)	P=0.54 (FDR=0.576)
Spleen	P=0.212 (FDR=0.287)	P=0.153 (FDR=0.237)
Stomach	P=0.08 (FDR=0.132)	P=0.082 (FDR=0.146)
Testis	P=0.304 (FDR=0.356)	P=0.242 (FDR=0.314)
Thyroid	P=0.008 (FDR=0.024)	P=0.025 (FDR=0.075)
Uterus	P=0.001 (FDR=0.004)	P=0.005 (FDR=0.018)
Vagina	P=0.027 (FDR=0.059)	P=0.068 (FDR=0.131)
Whole Blood	P=0.001 (FDR=0.004)	P=0.002 (FDR=0.014)

Table S4.

Top archaic GEAs with strong signals of selection.

Loci exceeding the length expected from incomplete lineage sorting (FDR<0.05, see Methods) for two estimates of the human mutation rate (μ_1=0.5x10^{-9} and μ_1=1x10^{-9} per basepair per year) are marked with "X" in the respective columns 4 and 5.

Archaic locus	Selection P-value	Genes with significant differential expression in the tissues in the top 5% GEA (direction of expression change for individuals with the Neandertal allele is shown as +/-)	μ_1: FDR <0.05	μ_2: FDR <0.05
chr11:102344970-102353988	8.7e-4	**TMEM123**: Colon - Transverse (+), Heart - Left Ventricle (+), Muscle - Skeletal (+)<br>**MMP7:** Nerve - Tibial (-), Vagina (+)	**X**	**X**
chr11:120084221-120124302	4.4e-4	**OAF**: Adipose - Visceral (Omentum) (+), Brain - Caudate (basal ganglia) (+), Brain – Cerebellum (+), Cells - Transformed fibroblasts (+), Muscle – Skeletal (-)<br>**AP000679.2**: Esophagus – Mucosa (-), Uterus (+)<br>**TRIM29**: Brain – Cortex (+), Pancreas (+), Small Intestine - Terminal Ileum (+), Whole Blood (+)<br>**POU2F3**: Esophagus – Muscularis (-), Heart - Left Ventricle (-)		
chr6:29275499-29401057	1.9e-5	**OR5V1**: Artery – Tibial (+), Brain - Substantia nigra (+), Heart - Atrial Appendage (+), Heart - Left Ventricle (+), Nerve – Tibial (+), Testis<br>**OR2H1**: Testis (-), Vagina (+)<br>**OR10C1**: Artery – Tibial (+), Lung (+)<br>**OR12D3**: Esophagus - Mucosa (+)<br>**OR11A1**: Brain - Putamen (basal ganglia) (+), Testis (-)<br>**OR14J1**: Heart - Left Ventricle (+), Nerve - Tibial (+)<br>**OR12D2**: Brain - Putamen (basal ganglia) (+)	**X**	**X**
chr12:113350796-113444418	1.1e-8	**OAS3**: Brain - Caudate (basal ganglia) (-), Brain – Hippocampus (-), Brain - Putamen (basal ganglia) (-), Cells - Transformed fibroblasts (-), Esophagus – Mucosa (-), Pancreas (+), Spleen (-), Vagina (+)<br>**OAS2**: Cells - Transformed fibroblasts (-), Thyroid (+)<br>**OAS1**: Adipose – Subcutaneous (+), Esophagus – Mucosa (-), Skin - Sun Exposed (Lower leg) (+), Spleen (-)<br>**RPH3A**: Artery – Tibial (+)	**X**	**X**

Table S5.

Overlap with genome-wide association studies.

Overlap of GEA loci associated with the top 1% of differentially expressed genes (A), and non-synonymous archaic alleles (B), with GWAS traits (GWAS with $P<1x10^{-8}$ are highlighted in yellow, top candidate GWAS with $1x10^{-8}<P<1x10^{-5}$ are shown in green). Loci exceeding the length expected from incomplete lineage sorting (FDR<0.05, see Methods) for two estimates of the human mutation rate (μ_1=0.5x10^{-9} and μ_1=1x10^{-9} per basepair per year) are marked with "X" in the respective columns 4 and 5.

A

**GWAS phenotype**	**Archaic locus**	**Gene and Tissue with top 1% GEA (direction of expression change for individuals with Neandertal allele)**	**μ_1: FDR <0.05**	**μ_2: FDR <0.05**
Venous thromboembolism [49]	chr1:169133273-169453703	**NME7:**<br>Colon – Sigmoid (+)<br>Lung (+)	**X**	**X**
Urinary metabolites [50]	chr19:33190467-33468482	**CEP89:**<br>Minor Salivary Gland (+)<br>Muscle - Skeletal (-)<br>Pituitary (+)<br>Testis (-)<br>Vagina (+)<br>**SLC7A9:**<br>Adipose - Subcutaneous (+)<br>Artery - Aorta (+)<br>Artery - Coronary (+)<br>Artery - Tibial (+)<br>Brain - Cerebellum (+)<br>Brain - Hippocampus (+)<br>Brain - Substantia nigra (+)<br>Breast - Mammary Tissue (+)<br>Colon - Sigmoid (+)<br>Colon - Transverse (+)<br>Esophagus - Gastroesophageal Junction (+)<br>Esophagus - Mucosa (+)<br>Esophagus - Muscularis (+)<br>Lung (+)<br>Minor Salivary Gland (+)<br>Muscle - Skeletal (+)<br>Nerve - Tibial (+)<br>Ovary (+)<br>Pancreas (+)<br>Pituitary (+)<br>Prostate (+)<br>Skin - Not Sun Exposed (Suprapubic) (+)<br>Skin - Sun Exposed (Lower leg) (+)<br>Stomach (+)<br>Testis (+)<br>Thyroid (+)<br>Uterus (+)<br>Vagina (+)<br>**TDRD12:**<br>Artery - Tibial (-)<br>Brain - Caudate (basal ganglia) (+)<br>Breast - Mammary Tissue (-)	**X**	**X**
LDL cholesterol, HDL cholesterol [51]	chr3:132119691-132421998	**DNAJC13:**<br>Brain - Putamen (basal ganglia) (-)	**X**	**X**
Butyrylcholinesterase levels [52]	chr3:165476224-165603543	**BCHE :**<br>Adipose - Subcutaneous (-)<br>Skin - Not Sun Exposed (Suprapubic) (-)<br>Thyroid (-)	**X**	**X**
Helicobacter pylori serologic status [53]<br>Self-reported allergy [54]	chr4:38763693-38906951	**FAM114A1:**<br>Adipose - Subcutaneous (+)<br>Breast - Mammary Tissue (+)<br>Cells - EBV-transformed lymphocytes (+)<br>Esophagus - Gastroesophageal Junction (+)<br>Esophagus - Mucosa (+)	**X**	**X**

		Lung (+)<br>Muscle - Skeletal (+)<br>Pituitary (+)<br>Skin - Sun Exposed (Lower leg) (+)<br>Spleen (+)<br>**TLR1:**<br>Brain - Hippocampus (+)<br>Cells - EBV-transformed lymphocytes (+)<br>Colon - Sigmoid (-)<br>Esophagus - Muscularis (-)<br>Liver (-)<br>Pituitary (+)<br>Stomach (-)<br>**TLR6:**<br>Adipose - Subcutaneous (-)<br>Breast - Mammary Tissue (-)<br>Cells - EBV-transformed lymphocytes (+)<br>Esophagus - Mucosa (-)<br>Muscle - Skeletal (-)<br>Pituitary (+)<br>Skin - Sun Exposed (Lower leg) (-)<br>Spleen (-)<br>**TLR10:**<br>Adrenal Gland (+)		
Pulmonary function [55]<br>Pulmonary function (interaction) [56]	chr4:106508526-106814302	**GSTCD:**<br>Pituitary (+)	**X**	**X**
Body mass index [36]	chr4:169300451-169340431	**DDX60L:**<br>Adipose - Subcutaneous (-)<br>Heart - Atrial Appendage (-)<br>Pituitary (-)	**X**	**X**
Other erythrocyte phenotypes [57]<br>Red blood cell traits [58]	chr6:25319900-25408104	**LRRC16A:**<br>Cells - Transformed fibroblasts (+)<br>Ovary (-)	**X**	**X**
Rheumatoid arthritis [59]	chr6:44228815-44282897	**NFKBIE:**<br>Colon - Transverse (-)<br>Pancreas (-)	**X**	**X**
Metabolite levels [60]	chr6:111415836-111951212	**KIAA1919**:<br>Adrenal Gland (-)<br>Colon - Sigmoid (-)<br>Esophagus – Muscularis (-)<br>Heart - Atrial Appendage (-)<br>Heart - Left Ventricle (-)<br>Liver (-)<br>Lung (-)<br>Pancreas (-)<br>Thyroid (-)<br>**SLC16A10:**<br>Brain - Anterior cingulate cortex (BA24) (-)	**X**	**X**
Acute lymphoblastic leukemia (B-cell precursor) [61]<br>Acute lymphoblastic leukemia (childhood) [62]	chr7:50482629-50575975	**DDC:**<br>Brain - Cerebellar Hemisphere (+)<br>Muscle - Skeletal (-)<br>Testis (+)	**X**	**X**
Systemic lupus erythematosus,<br>Primary biliary cirrhosis,<br>Systemic sclerosis,<br>Rheumatoid arthritis,<br>Celiac disease and Rheumatoid arthritis,<br>Multiple sclerosis,<br>Systemic lupus erythematosus and Systemic sclerosis [26-35]	chr7:128585616-128710302	**TNPO3:**<br>Brain - Caudate (basal ganglia) (+)	**X**	**X**
Crohn`s disease and psoriasis [63]<br>Crohn`s disease [23]	chr10:64387934-64447352	**ZNF365:**<br>Cells - EBV-transformed lymphocytes (+)		**X**
Parkinson`s disease [37]	chr10:71580120-71591034	**COL13A1:**<br>Brain - Cerebellum (-)		
Blood pressure [64]<br>Interleukin levels (IL10, IL1Ra, IL6), in plasma [65]	chr11:9374407-9498746	**IPO7:**<br>Cells - EBV-transformed lymphocytes (-)	**X**	**X**
Interleukin-18 levels [24]<br>Inflammatory biomarkers [25]	chr11:112005483-112084109	**IL18:**<br>Lung (-)<br>Pancreas (-)<br>Skin - Sun Exposed (Lower leg) (-)	**X**	**X**
Height [66]	chr12:56623347-56753822	**STAT2:**<br>Liver (+)	**X**	**X**
Systemic lupus erythematosus [67]	chr12:129271719	**SLC15A4:**	**X**	**X**

	-129330378	Artery - Aorta (+)<br>Testis (-)		
Schizophrenia [68]	chr15:84641125-85431566	**ADAMTSL3:**<br>Brain - Amygdala (-)	**X**	**X**
Dehydroepiandrosterone sulphate levels [69]	chr19:48332914-48465312	SULT2A1:<br>Adrenal Gland (-)	**X**	**X**

B

**GWAS phenotype**	**Non-synonymous archaic ALLELE**	**Gene**	**μ_1: FDR<0.05**	**μ_2: FDR<0.05**
Rheumatoid arthritis [59]	chr6:44232977	**NFKBIE**	**X**	**X**
Metabolite levels [60]	chr6:111628626	**REV3L**	**X**	**X**
Crohn`s disease [23]	chr10:64415184	**ZNF365**		**X**
Acenocoumarol maintenance dosage [70]	chr10:96798749	**CYP2C8**	**X**	**X**
Height [66]	chr12:56740682	**STAT2**		
Stroke [71]	chr14:61924239	**PRKCH**	**X**	**X**
Schizophrenia [68]	chr15:84706461	**ADAMTSL3**	**X**	**X**
Urinary metabolites [50]	chr19:33309030	**TDRD12**	**X**	**X**
Prostate-specific antigen levels [72]	chr19:51361757	**KLK3**		

Table S6.

Frequency (%) of tag Neandertal allele in Europeans and Asians for GWAS and selection candidate loci

locus		chr12:113350796-113444418	chr4:169300451-169340431	chr10:71580120-71591034	chr7:128585616-128710302	chr10:64387934-64447352	chr11:112005483-112084109
gene(s)		*OAS1/OAS2/OAS3*	*DDX60L*	*COL13A1*	*TNPO3*	*ZNF365*	*IL18*
tag allele		chr12:113366899	chr4:169330384	chr10:71580120	chr7:128617466	chr10:64415184	chr11:112023827
Europe	CEU	37.9	12.6	8.7	11.1	23.7	22.7
	TSI	44.4	13.9	9.1	8.8	23.6	16.7
	FIN	27.8	20.7	7.4	11.6	27.3	23.2
	GBR	36.4	14.1	11.6	10.3	31.5	24.5
	IBS	36.0	15.4	15.3	8.9	30.4	22.9
East Asia	CHB	30.6	11.2	1.9	0.5	7.3	0.5
	JPT	15.9	11.5	2.1	0	7.7	0.5
	CHS	20.8	14.4	5.2	0	11.6	0
	CDX	25.3	10.6	5.3	0	14.1	0.5
	KHV	34.7	10.4	3.4	0.5	13.4	0
South Asia	GIH	29.7	7.5	10.1	14.6	14.2	11.3
	PJL	34.9	14.6	9.3	21.9	13.0	5.2
	BEB	19.2	11.6	11.1	8.1	12.2	5.2
	STU	27.7	11.7	8.2	12.6	12.6	3.8
	ITU	29.1	10.7	9.3	9.2	12.6	4.4
