# Supplementary material for: Functional implications of Neandertal introgression in modern humans
Source: Genome Biol. 2017 Apr 3;18:61. doi: 10.1186/s13059-017-1181-7 (PMC5376702; doi:10.1186/s13059-017-1181-7)
Supplement: Supplementary file 2 — Supplementary figures. (PDF 719 kb) [file 13059_2017_1181_MOESM2_ESM.pdf]

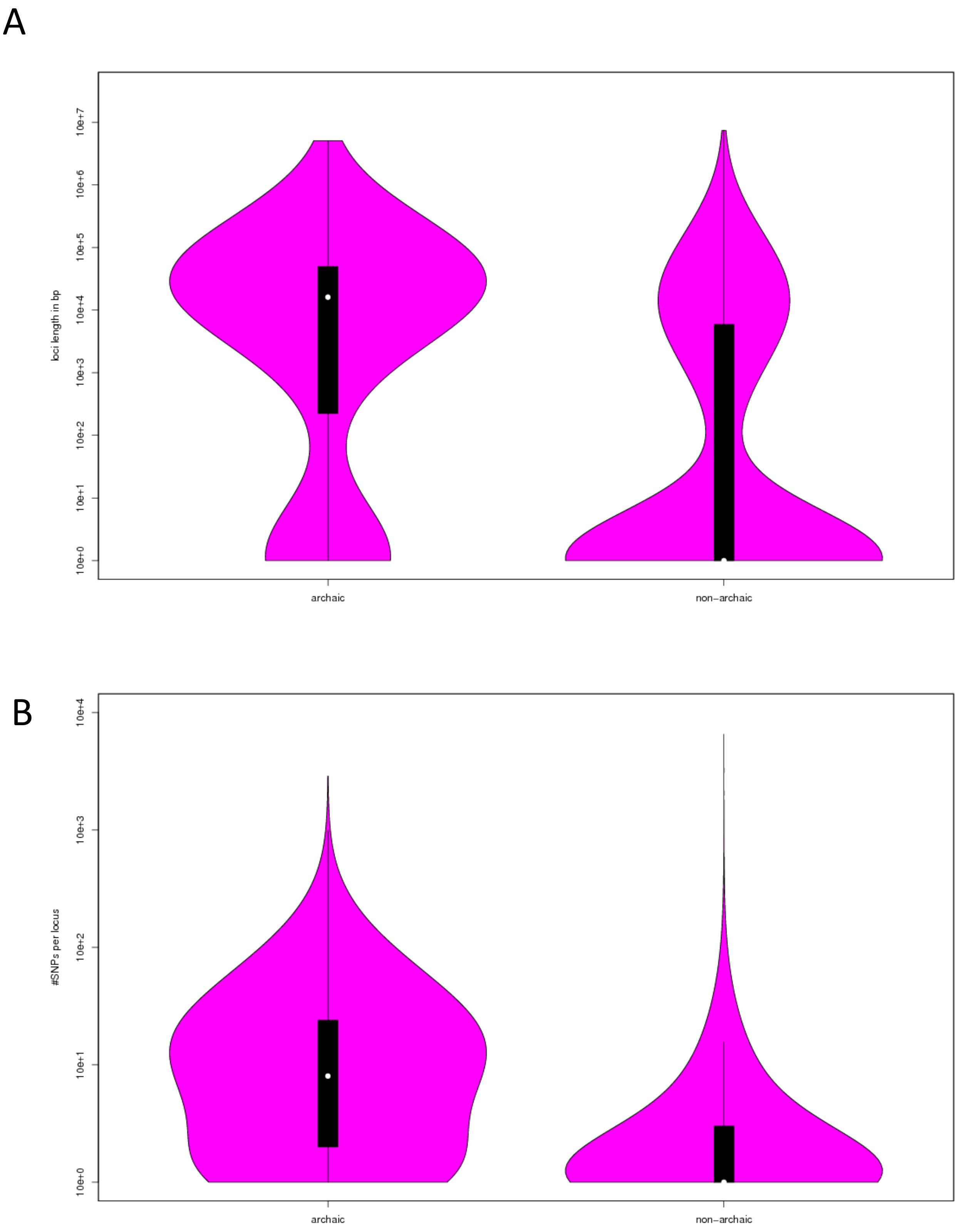

Figure S1. The lengths (A) and number of SNPs (B) for archaic and non-archaic loci. **(A)** Maximal distance between alleles in LD (r^2>0.8). For visualization purposes we define the length of a locus with a single SNP to be 1 base pair. (**B**) The number of SNPs within archaic and non-archaic loci.

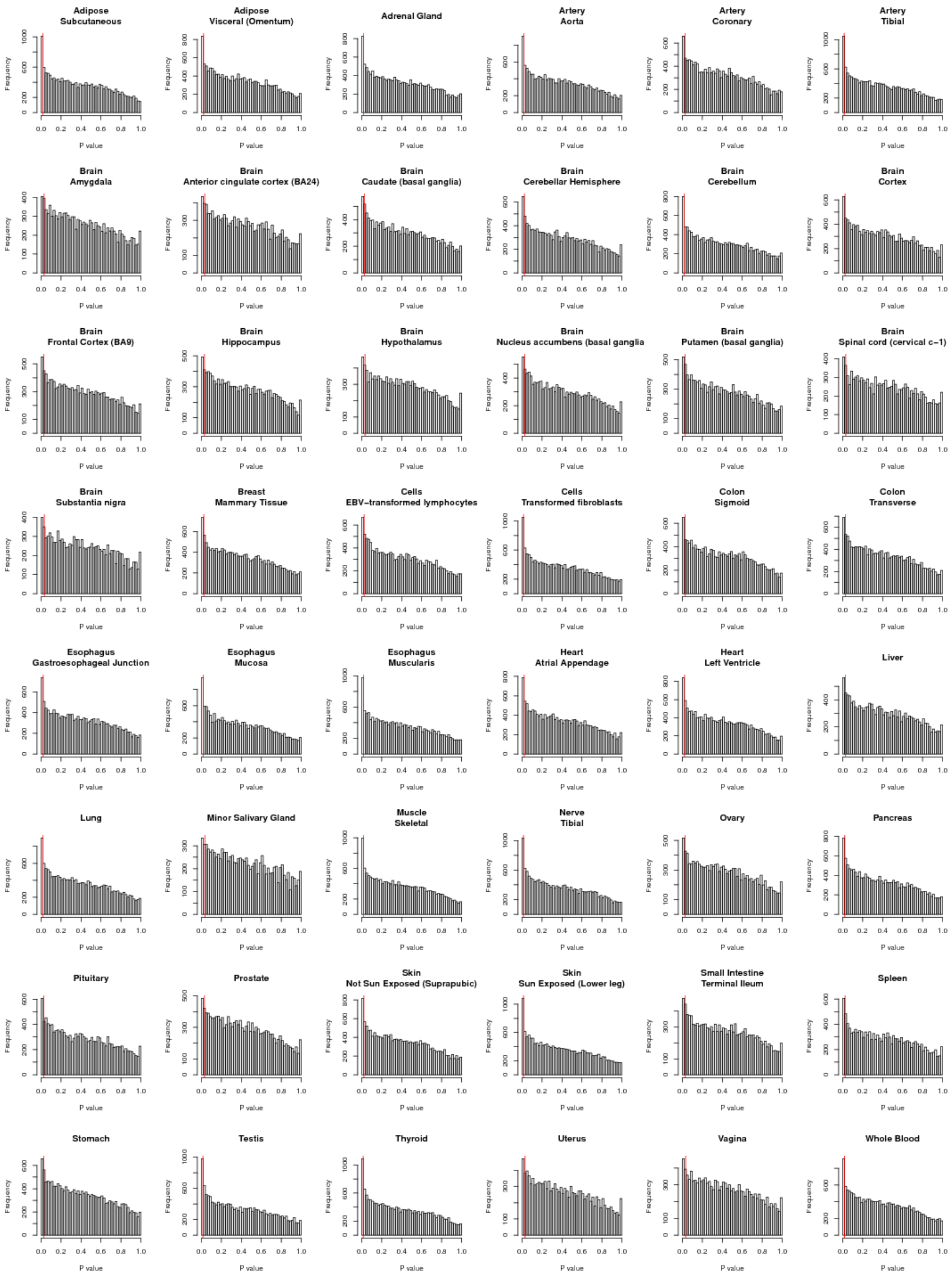

Figure S2. Distribution of p-values for archaic loci in 48 tissues. The 5% quantile is shown as a red line.

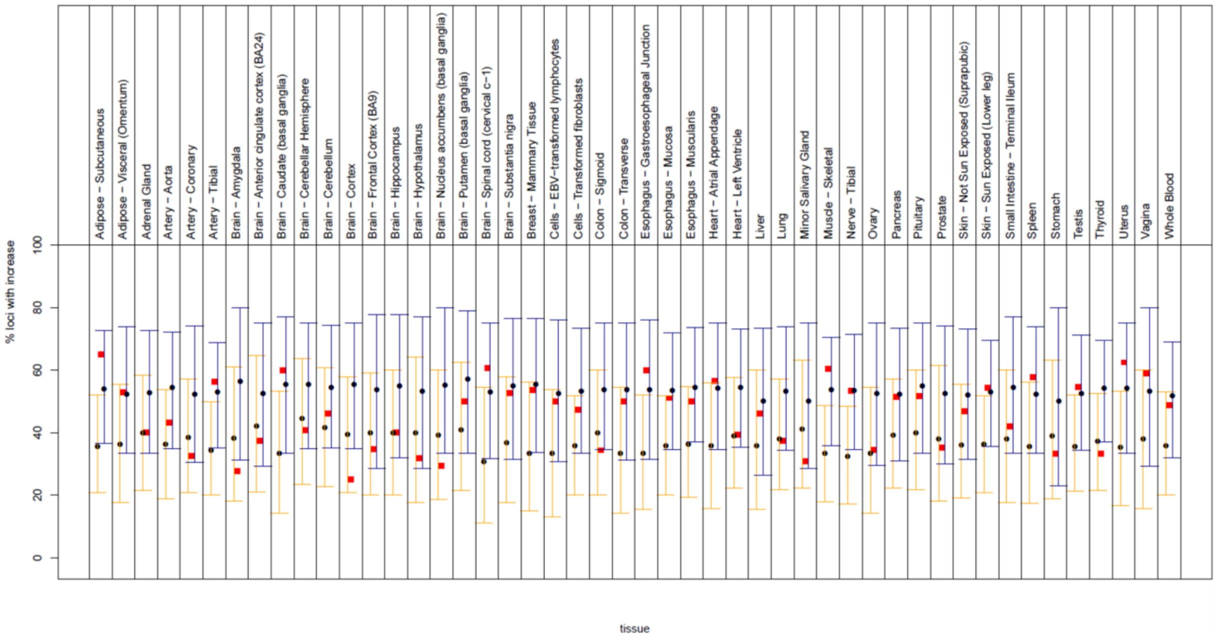

Figure S3. Direction of change during recent modern human history for alleles showing significant frequency changes in individual tissues. We calculate the percentage of archaic alleles showing association with differential expression in each tissue that increase in frequency, and compare to two sets of frequency matched alleles : (i) archaic alleles not associated with differential expression and (ii) non-archaic alleles that are associated with differential expression.

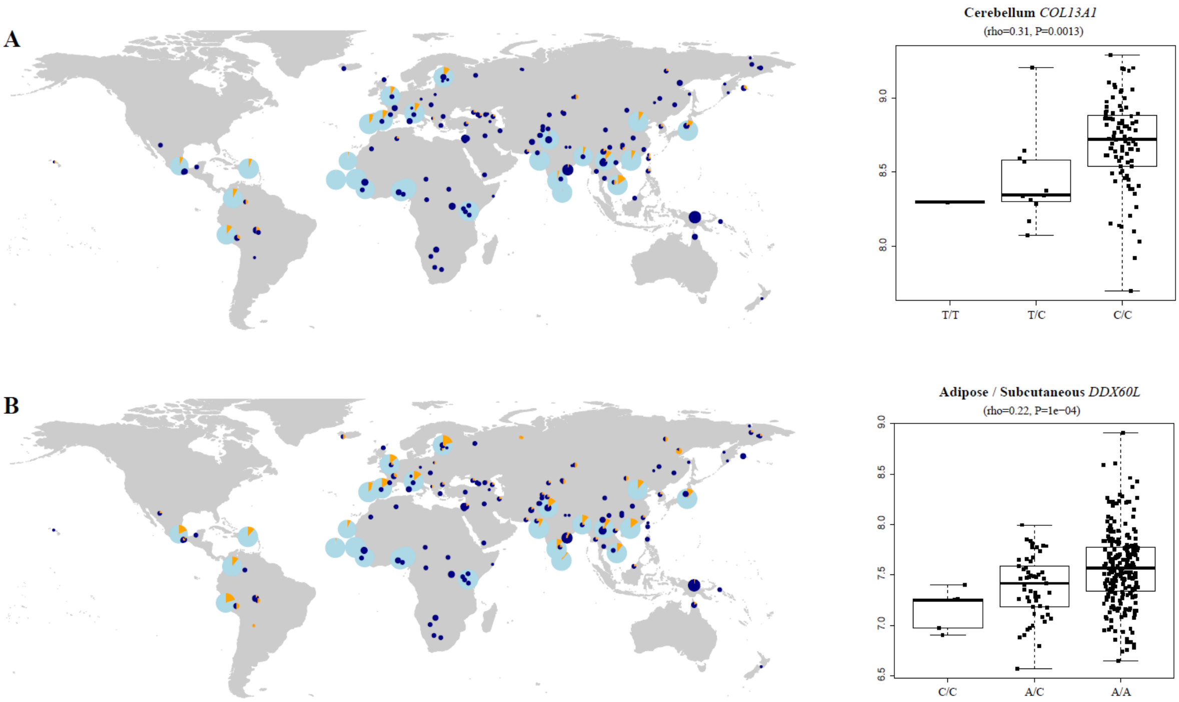

Figure S4. Global frequency distribution and differential expression of archaic alleles at *DDX60L* and *COL13A1*

Left: The frequency of the archaic locus (orange) spanning *COL13A1* (A) and *DDX60L* (B) in present day human populations from the 1,000 Genomes phase III (light-blue) and Simons Genome Diversity Project (dark-blue) datasets. The sizes of the pie charts are proportional to the number of individuals per population.

Right: The expression (y-axis, log-transformed read counts) for *COL13A1* (A) in cerebellum and for *DDX60L* (B) in subcutaneous adipose tissue dependent on the genotypes. The expression distributions for both homozygote and the heterozygote states are shown as boxplots and expression values for all individuals are shown as black squares. The Spearman correlation coefficient and the corresponding P-value for both tissues are shown above each graph. The introgressed Neandertal alleles are “C” in both cases (*COL13A1*: chr10:71580120; *DDX60L*: chr4:169330384).
